# Structural models of human ACE2 variants with SARS-CoV-2 Spike protein for structure-based drug design

**DOI:** 10.1038/s41597-020-00652-6

**Published:** 2020-09-16

**Authors:** Marija Sorokina, João M. C. Teixeira, Susana Barrera-Vilarmau, Reinhard Paschke, Ioannis Papasotiriou, João P. G. L. M. Rodrigues, Panagiotis L. Kastritis

**Affiliations:** 1 Institute of Biochemistry and Biotechnology, Martin Luther University Halle-Wittenberg, Kurt-Mothes-Str. 3, 06120 Halle/Saale, Germany; 2 RGCC International GmbH, Baarerstrasse 95, Zug, 6300 Switzerland; 3 BioSolutions GmbH, Weinbergweg 22, 06120 Halle/Saale, Germany; 4 Program in Molecular Medicine, Hospital for Sick Children, Toronto, Ontario M5G 0A4 Canada; 5 Institute of Advanced Chemistry of Catalonia (IQAC), CSIC, Jordi Girona, 18-26, 08034 Barcelona, Spain; 6 Biozentrum, Martin Luther University Halle-Wittenberg, Weinbergweg 22, 06120 Halle/Saale, Germany; 7 Department of Structural Biology, Stanford University, Stanford, CA 94305 USA; 8 Interdisciplinary Research Center HALOmem, Charles Tanford Protein Center, Martin Luther University Halle-Wittenberg, Kurt-Mothes-Str. 3a, 06120 Halle/Saale, Germany

**Keywords:** Data mining, Protein structure predictions, Computational models

## Abstract

Emergence of coronaviruses poses a threat to global health and economy. The current outbreak of SARS-CoV-2 has infected more than 28,000,000 people and killed more than 915,000. To date, there is no treatment for coronavirus infections, making the development of therapies to prevent future epidemics of paramount importance. To this end, we collected information regarding naturally-occurring variants of the Angiotensin-converting enzyme 2 (ACE2), an epithelial receptor that both SARS-CoV and SARS-CoV-2 use to enter the host cells. We built 242 structural models of variants of human ACE2 bound to the receptor binding domain (RBD) of the SARS-CoV-2 surface spike glycoprotein (S protein) and refined their interfaces with HADDOCK. Our dataset includes 140 variants of human ACE2 representing missense mutations found in genome-wide studies, 39 mutants with reported effects on the recognition of the RBD, and 63 predictions after computational alanine scanning mutagenesis of ACE2-RBD interface residues. This dataset will help accelerate the design of therapeutics against SARS-CoV-2, as well as contribute to prevention of possible future coronaviruses outbreaks.

## Abstract (summary)

**Table:** 

Measurement(s)	Molecular Genetic Variation
Technology Type(s)	digital curation
Factor Type(s)	ACE2 variants
Sample Characteristic - Organism	Homo sapiens

Machine-accessible metadata file describing the reported data: 10.6084/m9.figshare.12902498

## Background & Summary

The novel and highly-pathogenic coronavirus (SARS-CoV-2) emerged from Wuhan city, Hubei province of China late 2019^1^, spreading rapidly across the world and causing a global public health emergency with more than 28,000,000 infections in more than 200 countries. Symptoms include dry cough, tiredness, fever as well as severe pneumonia with additional extrapulmonary manifestations and complications^2^.

SARS-CoV-2 is the latest member of the betacoronavirus genus which includes SARS-CoV, MERS-CoV, bat SARS-related coronaviruses (SARSr-CoV), as well as others infecting diverse animal species and humans^3^. Although bat coronavirus RaTG13 seems to be the closest relative of the SARS-CoV-2, sharing > 93% sequence identity in the spike (S) gene, SARS-CoV and other SARSr-CoVs are distinct with < 80% sequence identity^4^. This (S) gene translates a protein which assembles in homotrimers on the viral envelope, forming the “corona” after which the group is named. Coronaviruses use this spike glycoprotein, composed of an S1 and an S2 subunit in each spike monomer, to bind host cell receptors^5^. This initial binding event triggers multiple events that culminate with the fusion of cell and viral membranes for cell entry. Recent studies have pointed the important and conserved role of the cell membrane receptor angiotensin-converting enzyme 2 (ACE2) in mediating entry of SARS-CoV-2^6^. It is known that SARS-CoV-2 spike interacts with ACE2 through a receptor binding domain (RBD), which binds ACE2 with low nM affinity^7^, and then induces dissociation of S1 with ACE2, prompting the S2 to transfer from a prefusion to a postfusion state essential for membrane fusion. Therefore, spike protein RBD binding to the ACE2 receptor is the first key-step which enables the virus to enter target cells.

Recent crystallographic and electron cryo-microscopic (cryo-EM) studies have provided details into the structure of the SARS-CoV-2 S protein, resolved in its free state in both closed and open conformations^8,9^, but also bound (the RBD domain) to the ACE2 membrane receptor^8,10,11^. The atomic-level structural information greatly improves our understanding of the interaction between SARS-CoV-2 and susceptible cells, providing a precise target for neutralizing antibodies, and assisting structure-based drug design - urgently needed in our ongoing combat against the virus. To our knowledge, all current structural studies have examined the interaction of the SARS-CoV-2 RBD only with the main ACE2 membrane receptor variant while studies probing SARS-CoV-2 RBD domain in complex with ACE2 variants are limited^12,13^.

Studying the effect of naturally-occurring single nucleotide polymorphisms (SNPs) of ACE2 in humans^12–14^ on its affinity to the SARS-CoV-2 RBD domain is necessary for the development of appropriate therapeutics. ACE2 variants are known to be related to cardiovascular disease^15^ and indeed, a large proportion of patients infected by SARS-CoV-2 have underlying cardiac risk factors^14,15^. Some of these ACE2 variants result in amino acid changes (missense mutations), which consequently affect the 3D structure of the formed complex. Further, some variants might promote different infectivity rates due to different affinities of ACE2 to the SARS-CoV-2 RBD domain.

Therefore, understanding variation of ACE2 in human population is of critical importance for the development of therapeutic strategies against coronaviruses. Despite *in vitro* studies on other ACE2 variants, there has not been a systematic study of the effects of these variations on the 3D structure of the protein and its complex with RBD. 3D models of ACE2 variants in complex with the SARS-CoV-2 RBD will be of use to industrial and academic communities alike because they can be starting points for drug design while providing further understanding into the recognition of SARS-CoV-2 S proteins by ACE2. To this end, we assembled a structure-based dataset of ACE2 variants in complex with the SARS-CoV-2 RBD, communicating in total 242 structural models.

## Methods

### Database search

**Fig. 1:**
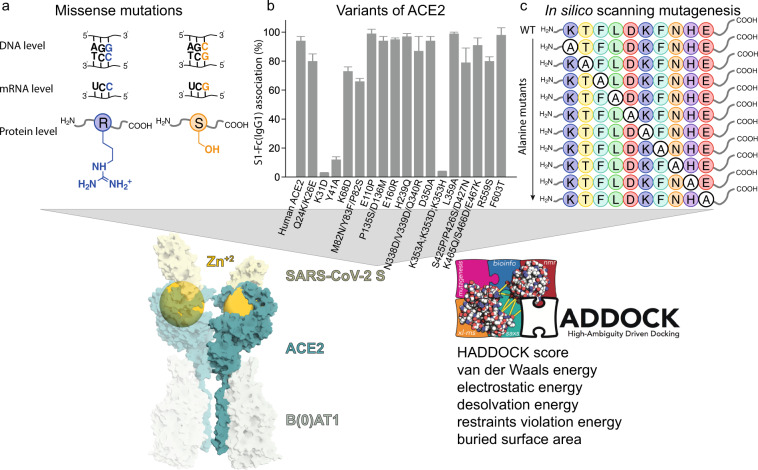
Schematic overview of the structure-based benchmark of ACE2 variants-S protein complexes. All available variants are collected from (**a**) missense mutations identified in the human genome; (**b**) overexpressed constructs of ACE2 variants reported in the literature; and (**c**) designed alanine scanning mutagenesis variants of ACE2, targeting the interface residues with the S protein (upper panels). In the bottom panel, a structure-based benchmark including all variants is assembled for use in drug development, and optimization of the interface of the variant by including the Zn^+2^ ion is performed using the HADDOCK software. Zn^2+^ is represented magnified, because it was considered for calculations.

To identify all relevant variants of ACE2, we performed a search in multiple databases and created workflow for assembling the variants in complex with the SARS-CoV-2 RBD **(**Fig. 1**)**. For variants naturally occurring in the human population, we searched gnomAD^16^ and identified 155 unique missense ACE2 variants, 140 of which are mapped on the structural model (Online-only Table 1). For variants of ACE2 with known binding data we searched Uniprot^17^ and the corresponding articles which describe site-directed mutagenesis experiments^18–20^. We identified 39 variants in total with 49 reported mutations **(**Table 1**)**.

**Table 1:** Mutations reported in the literature mappable on the cryo-EM structure.

#N	Variation	Haddock Score	vdW (a.u.)	Elec (a.u.)	Desol (a.u.)	BSA (Å^2^)	Effect
0	WT	−89.9 ± 5.2	−54.7 ± 2.4	−184.5 ± 9.3	1.7 ± 4	1729 ± 34.6	WT
1	QAK24-26KAE	−95.2 ± 4.1	−52 ± 2.2	−196 ± 33	−4 ± 5.1	1709.5 ± 68.2	B
2	K31D	−85.7 ± 6.5	−56.4 ± 2.5	−176.9 ± 11.6	6 ± 6.4	1692.6 ± 10.3	D
3*	E37A	−91.2 ± 3.7	−52.2 ± 1.1	−176.9 ± 30.6	−3.6 ± 7	1660.4 ± 22.9	A
4*	D38A	−86 ± 8.3	−55.5 ± 2.2	−174.8 ± 33.8	4.5 ± 4.2	1685.6 ± 53.2	A
5*	Y41A	−92.1 ± 4.3	−52.7 ± 1.7	−171.7 ± 17	−5 ± 6.9	1687.3 ± 26.7	C
6	K68D	−85.9 ± 5.9	−55.5 ± 1.8	−188.8 ± 21	7.4 ± 4.6	1739.4 ± 52	B
7	MYP82-84NFS	−99 ± 6.4	−53.9 ± 2.6	−176.8 ± 11.8	−9.7 ± 7.4	1750 ± 36.3	E
8	E110P	−90.2 ± 3.2	−56.2 ± 2.3	−208.3 ± 12.1	7.6 ± 6.3	1739.8 ± 32.7	A
9	PD135-136SM	−102 ± 3.7	−56.6 ± 2.1	−210.3 ± 19.8	−3.4 ± 8.8	1774 ± 53.6	A
10	E160R	−90.5 ± 2	−55.5 ± 2.5	−182 ± 12.4	1.4 ± 5.7	1721 ± 30.4	A
11	R169Q	−98 ± 4.2	−53.5 ± 2.9	−202 ± 22.8	−4.1 ± 2.5	1733.7 ± 65.7	F
12	R192D	−111.8 ± 5	−58.2 ± 2.7	−198.3 ± 11.3	−13.9 ± 6.4	1764.1 ± 12.6	A
13	R219D	−94.4 ± 4.9	−54.6 ± 4.6	−206 ± 11.9	1.3 ± 5.5	1756.8 ± 25.6	A
14	H239Q	−97.1 ± 8.2	−56 ± 1	−194.1 ± 16.6	−2.3 ± 9.4	1697.8 ± 39.8	A
15	W271Q	−95.5 ± 3.2	−54.8 ± 1.4	−208.4 ± 8.7	1 ± 4.5	1736.5 ± 15	F
16	R273Q	−104.1 ± 6.3	−55.7 ± 2.1	−215.2 ± 17.4	−5.4 ± 6.1	1767.5 ± 38.9	G
17	K309D	−91.1 ± 7.8	−57.8 ± 2.2	−180.4 ± 17.5	2.9 ± 9.2	1759.9 ± 32	A
18	E312A	−90.9 ± 2.5	−57 ± 1.9	−172.6 ± 15.8	0.7 ± 3.2	1705.4 ± 23	A
19*	T324A	−99.7 ± 4.7	−54.9 ± 0.8	−189.8 ± 26.4	−6.9 ± 3.9	1726.7 ± 7 ± 1	A
20	NVQ338-340DDR	−84 ± 3.7	−54 ± 3.9	−193.2 ± 10.9	8.6 ± 4.5	1730.3 ± 28.2	A
21	H345A	−92.3 ± 4.8	−55 ± 0.8	−188.4 ± 4.7	0.4 ± 4.9	1674.9 ± 26.7	G
22*	D350A	−95.6 ± 7.5	−54.7 ± 3.8	−184 ± 20.2	−4.1 ± 1.2	1691.8 ± 47.5	A
23	K353H	−104.7 ± 4.6	−57.2 ± 1.5	−174.6 ± 5.1	−12.7 ± 5.1	1749.2 ± 38.3	D
24*	K353A	−93 ± 1.9	−50.7 ± 3	−160 ± 34.1	−10.3 ± 4.7	1668.1 ± 28.4	D
25	K353D	−101.9 ± 8	−53.8 ± 3.3	−201 ± 8.3	−7.9 ± 6.6	1739 ± 30.3	D
26*	D355A	−96.4 ± 2	−53.9 ± 1.9	−207.4 ± 8	−1 ± 2.8	1719 ± 16.3	C
27*	R357A	−101.9 ± 5.2	−58.3 ± 0.9	−199.5 ± 11.7	−3.7 ± 6.1	1759.3 ± 41.2	C
28	L359K	−98.8 ± 3.9	−53.9 ± 4.2	−174 ± 27.4	−10 ± 2.1	1728.7 ± 32.5	A
29	L359A	−90.9 ± 5.6	−54 ± 1.2	−205.2 ± 17.2	4.2 ± 7.7	1733.2 ± 38.9	A
30*	M383A	−105.6 ± 4.7	−59.6 ± 3.1	−197.5 ± 13.7	−6.5 ± 7.5	1788.8 ± 25.8	B
31*	P389A	−104.3 ± 9.9	−55.6 ± 5.2	−198 ± 5.9	−9.1 ± 9.3	1755.2 ± 61	B
32*	R393A	−106.3 ± 1	−54.5 ± 3.4	−201.1 ± 10.8	−11.5 ± 3	1715.3 ± 43.5	B
33	SPD425-427PSN	−96.7 ± 3.9	−55.7 ± 0.2	−171.9 ± 14.3	−6.7 ± 2.7	1727.6 ± 53	B
34	KGE465-467QDK	−81.8 ± 3.1	−53.1 ± 3.7	−184.4 ± 23.9	8.2 ± 3	1753.7 ± 38.8	A
35	K481Q	−97.1 ± 3.4	−56.8 ± 1.6	−198.8 ± 9.3	−0.6 ± 1.6	1726 ± 42.2	H
36	H505A	−99.7 ± 7.8	−55.4 ± 3	−193.6 ± 12.2	−5.6 ± 7.1	1724.3 ± 26.3	G
37	R514Q	−87.6 ± 2.5	−54.2 ± 2.5	−193.1 ± 16.5	5.2 ± 3.8	1739.2 ± 36.7	H
38	R559S	−91.4 ± 7.5	−54.6 ± 1.4	−203.1 ± 14.5	3.7 ± 7.7	1744.3 ± 12.4	B
39	F603T	−92.6 ± 3.3	−58 ± 1.7	−165.9 ± 16.3	−1.4 ± 3.3	1739.6 ± 24.7	A

**Fig. 2:**
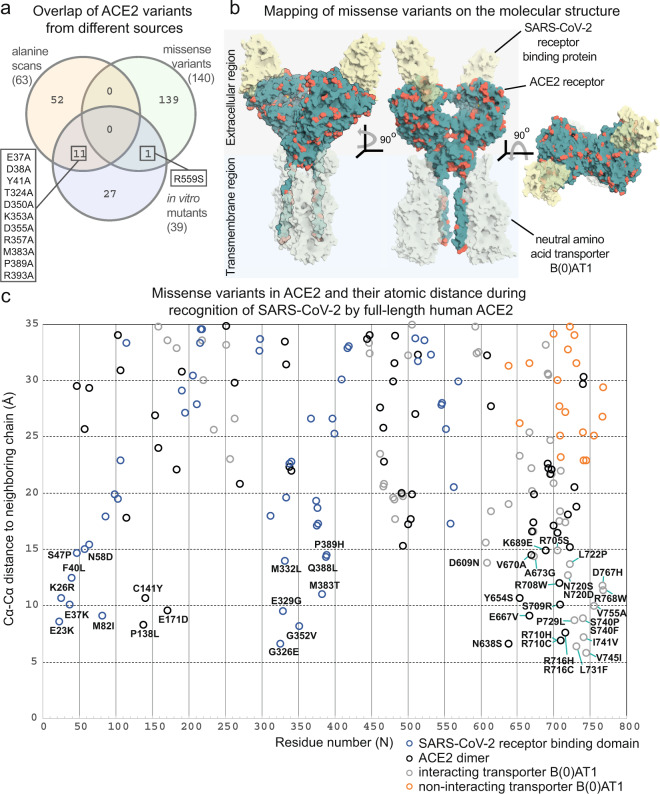
Overview of the datasets used in the study with a focus on the localization of naturally occurring ACE2 variants in the human population. (**a**) Venn diagram showing the variability of sequence variants among the 3 different datasets assembled in this study. (**b**) structure-based mapping of missense variants on the wild-type ACE2 in complex with the SARS-CoV-2 RBD, in the presence of the B^0^AT1 complex^11^. Variants are distributed on the surface of the complex. (**c**) Measurement of distances of all mappable missense variants and report of the variants close to the different interfaces identified in the cryo-EM model of ACE2 in complex with the SARS-CoV-2 RBD, in the presence of the B^0^AT1 complex^11^.

Table includes energetic calculations with HADDOCK. vdW – van der Waals interaction score; Elec – electrostatic interaction score; Desol – desolvation score; BSA – buried surface area; a.u. – arbitrary units of energy; Effect – The measured biochemical effect on the interaction with the SARS-CoV spike glycoprotein. A-No effect on interaction with SARS-CoV spike glycoprotein; B-Slightly inhibits interaction with SARS-CoV spike glycoprotein; C-Strongly inhibits interaction with SARS-CoV spike glycoprotein.; D-Abolishes interaction with SARS-CoV spike glycoprotein; E-Inhibits interaction with SARS-CoV spike glycoprotein.; F-About 95% loss of angiotensin I cleavage; G-Complete loss of enzyme activity; H- more than 50% loss of angiotensin I cleavage. *also included in the Alanine scanning mutagenesis data (see also Fig. 2a).

### Initial model and formation of variants

We used the cryo-EM model of the wild-type ACE2 in complex with the SARS-CoV-2 RBD, in the presence of the B°AT1 complex^11^ as a starting structure. Then, we systematically modelled all known variants of ACE2 and constructed the equivalent 2019-nCoV RBD/ACE2-B°AT1 complexes using PyMOL^21^ and the “mutagenesis” wizard. We picked the rotamers with the lowest clash score and stored the models in both *.pdb* and *.cif* formats. These models include all co-factors, namely ions and structurally-important glycan molecules that were structurally resolved^11^.

### *In silico* alanine scanning mutagenesis

The initial model^11^ was used to calculate interface residues by considering all residue-residue pairs of the wild-type ACE2 and the SARS-CoV-2 RBD within 10 Å distance of each other. These positions were then individually mutated to alanine residues (Ala). In total, we selected 63 residues, plus 6 which were alanine residues in the wild-type sequence and served as positive controls **(**Table 2**)**.

**Table 2:** Computationally-generated alanine scanning mutagenesis variants.

#N	Variation	Haddock Score	vdW (a.u.)	Elec (a.u.)	Desol (a.u.)	BSA (Å^2^)
0	WT	−89.9 ± 5.2	−54.7 ± 2.4	−184.5 ± 9.3	1.7 ± 4	1729 ± 34.6
1	I21A	−90.8 ± 4.8	−54.4 ± 3.2	−181.3 ± 10.7	−0.1 ± 6.1	1720.2 ± 22.4
2	E22A	−95.5 ± 5.5	−56.2 ± 1.6	−182.9 ± 11.8	−2.7 ± 9.4	1728.6 ± 51.1
3	E23A	−101 ± 6.2	−53.9 ± 2.1	−190.3 ± 20	−9.1 ± 4.7	1717.7 ± 13.1
4	Q24A	−93.4 ± 4	−52.5 ± 1.3	−203.1 ± 13.8	−0.3 ± 2.6	1694.3 ± 13.6
5*	A25A	−89.9 ± 5.2	−54.7 ± 2.4	−184.5 ± 9.3	1.7 ± 4	1729 ± 34.6
6	K26A	−101 ± 3.7	−57.4 ± 1.1	−207.2 ± 14.2	−2.2 ± 6.2	1791.8 ± 16.7
7	T27A	−92.1 ± 2.8	−51.8 ± 4.8	−192.7 ± 17	−1.8 ± 5.5	1718 ± 37.5
8	F28A	−84.9 ± 2.8	−51.4 ± 1.8	−218.9 ± 25.1	10.4 ± 5.4	1667.6 ± 34.8
9	L29A	−95.7 ± 5.9	−56 ± 2	−201.1 ± 17.4	0.5 ± 5.2	1751.6 ± 24
10	D30A	−77.4 ± 9	−53 ± 4.3	−108.9 ± 11.5	−2.6 ± 9.7	1693.3 ± 52.6
11	K31A	−89.4 ± 3.2	−51.6 ± 1.7	−164.1 ± 17.9	−4.9 ± 5.1	1662.1 ± 16.6
12	F32A	−92.8 ± 5.3	−55.2 ± 2.1	−188.6 ± 19.9	0.1 ± 5.5	1757.4 ± 30.6
13	N33A	−101.1 ± 4.2	−60 ± 5.1	−192 ± 21.6	−2.7 ± 6.3	1781.6 ± 45.9
14	H34A	−84.8 ± 5.3	−54.1 ± 3.8	−170.7 ± 27	3.5 ± 7.3	1639.3 ± 40.7
15	E35A	−86.3 ± 5.9	−55.3 ± 3.4	−183.1 ± 5.1	5.7 ± 4.2	1731.6 ± 12.6
16*	A36A	−89.9 ± 5.2	−54.7 ± 2.4	−184.5 ± 9.3	1.7 ± 4	1729 ± 34.6
17	E37A	−91.2 ± 3.7	−52.2 ± 1.1	−176.9 ± 30.6	−3.6 ± 7	1660.4 ± 22.9
18	D38A	−86 ± 8.3	−55.5 ± 2.2	−174.8 ± 33.8	4.5 ± 4.2	1685.6 ± 53.2
19	L39A	−95 ± 3.5	−55.1 ± 4.1	−187.6 ± 8.7	−2.3 ± 7.8	1714.8 ± 43.2
20	F40A	−100.5 ± 2.8	−55.5 ± 1.6	−202.6 ± 11.7	−4.5 ± 3.5	1774.9 ± 19.1
21	Y41A	−92.1 ± 4.3	−52.7 ± 1.7	−171.7 ± 17	−5 ± 6.9	1687.3 ± 26.7
22	Q42A	−94 ± 1.4	−57 ± 1.1	−174.8 ± 27.9	−2 ± 7.7	1726.6 ± 24.9
23	S43A	−96.1 ± 5.7	−53.4 ± 1.8	−194.9 ± 13.6	−3.7 ± 7.3	1722.4 ± 18.5
24	S44A	−96.7 ± 6.5	−54.8 ± 4.6	−195.9 ± 13	−2.7 ± 7.4	1759.8 ± 54.4
25	L45A	−92.4 ± 8.9	−52.5 ± 1.4	−179.1 ± 19.3	−4.1 ± 6.5	1700.4 ± 32.6
26*	A46A	−89.9 ± 5.2	−54.7 ± 2.4	−184.5 ± 9.3	1.7 ± 4	1729 ± 34.6
27	W48A	−103.2 ± 4.1	−56.1 ± 2.5	−196.2 ± 30.2	−7.8 ± 3.8	1753.5 ± 15.3
28	N49A	−106.7 ± 6.5	−55.7 ± 2.3	−206.7 ± 14.9	−9.6 ± 5.7	1760.6 ± 33.3
29	F72A	−92.9 ± 2.2	−55.4 ± 1.7	−188.7 ± 5.9	0.2 ± 2.8	1703.6 ± 33.8
30	E75A	−93.6 ± 3.8	−56.7 ± 2.9	−198.6 ± 22.3	2.8 ± 7	1762.6 ± 54.2
31	Q76A	−93.2 ± 2	−54.3 ± 1.5	−167.8 ± 31.8	−5.3 ± 4.3	1746.5 ± 39.6
32	S77A	−95.5 ± 5.5	−58.4 ± 2.8	−186.6 ± 16.5	0.2 ± 4.9	1737.3 ± 27.5
33	T78A	−99 ± 4.1	−55.2 ± 2	−190.8 ± 38.5	−5.6 ± 3.6	1795.4 ± 28.9
34	L79A	−91.5 ± 2	−57.3 ± 1.1	−171.1 ± 26.9	0 ± 6.6	1702.9 ± 23.3
35*	A80A	−89.9 ± 5.2	−54.7 ± 2.4	−184.5 ± 9.3	1.7 ± 4	1729 ± 34.6
36	Q81A	−100.3 ± 2.5	−53.7 ± 3.3	−192.3 ± 21.7	−8.1 ± 1.6	1748.7 ± 31
37	M82A	−85.9 ± 1.4	−52.6 ± 2	−172.6 ± 15.6	1.2 ± 4.2	1699.5 ± 41.1
38	Y83A	−90.5 ± 5.8	−53 ± 1.4	−207.8 ± 16.8	4.1 ± 6.4	1715.2 ± 35.1
39	P84A	−99 ± 2.1	−56.1 ± 2.6	−181.2 ± 25.8	−6.6 ± 5.4	1735 ± 24.9
40	M323A	−100.5 ± 3.9	−57 ± 2.7	−203.4 ± 19.4	−2.9 ± 1.5	1744.1 ± 11.5
41	T324A	−99.7 ± 4.7	−54.9 ± 0.8	−189.8 ± 26.4	−6.9 ± 3.9	1726.7 ± 71
42	Q325A	−94.3 ± 2	−57.7 ± 1.7	−193.5 ± 42.7	2.1 ± 8	1744 ± 44.3
43	G326A	−93.8 ± 1.8	−54.8 ± 1.4	−198.4 ± 26.4	0.7 ± 6.4	1775.1 ± 18.9
44	F327A	−100 ± 5.6	−55.9 ± 3.7	−170.8 ± 26.5	−9.9 ± 8.2	1730.1 ± 44.5
45	W328A	−102.9 ± 6	−55.5 ± 1.5	−188.5 ± 27.7	−9.7 ± 7.4	1711.5 ± 9.6
46	E329A	−91.3 ± 4.5	−56.7 ± 2.1	−186.4 ± 16.9	2.6 ± 4.7	1763.7 ± 24.1
47	N330A	−102 ± 5.8	−53.1 ± 1.9	−196 ± 8.2	−9.7 ± 2.4	1669.9 ± 9.8
48	S331A	−101 ± 4.1	−53.4 ± 1.4	−180.2 ± 12.9	−11.6 ± 2.7	1717 ± 27.6
49	D350A	−95.6 ± 7.5	−54.7 ± 3.8	−184 ± 20.2	−4.1 ± 1.2	1691.8 ± 47.5
50	L351A	−98.3 ± 7.2	−56.6 ± 3.5	−192.1 ± 20.4	−3.2 ± 7.6	1765.4 ± 23.3
51	G352A	−102.9 ± 1.9	−51.8 ± 2.7	−185.9 ± 17	−13.9 ± 4.1	1721 ± 53.6
52	K353A	−93 ± 1.9	−50.7 ± 3	−160 ± 34.1	−10.3 ± 4.7	1668.1 ± 28.4
53	G354A	−104.2 ± 1.8	−57.3 ± 2	−179.6 ± 43.2	−11 ± 8.7	1762.1 ± 37.1
54	D355A	−96.4 ± 2	−53.9 ± 1.9	−207.4 ± 8	−1 ± 2.8	1719 ± 16.3
55	F356A	−92.6 ± 3.2	−55.5 ± 1.4	−198.8 ± 14.7	2.7 ± 2.5	1736 ± 24.2
56	R357A	−101.9 ± 5.2	−58.3 ± 0.9	−199.5 ± 11.7	−3.7 ± 6.1	1759.3 ± 41.2
57	M383A	−105.6 ± 4.7	−59.6 ± 3.1	−197.5 ± 13.7	−6.5 ± 7.5	1788.8 ± 25.8
58	Y385A	−85.8 ± 5.9	−52 ± 3.8	−192.5 ± 12.1	4.7 ± 7.3	1718.9 ± 26.1
59*	A386A	−89.9 ± 5.2	−54.7 ± 2.4	−184.5 ± 9.3	1.7 ± 4	1729 ± 34.6
60*	A387A	−89.9 ± 5.2	−54.7 ± 2.4	−184.5 ± 9.3	1.7 ± 4	1729 ± 34.6
61	Q388A	−93.1 ± 4.6	−53.9 ± 4	−168.1 ± 10.2	−5.6 ± 4.8	1679.8 ± 43.6
62	P389A	−104.3 ± 9.9	−55.6 ± 5.2	−198 ± 5.9	−9.1 ± 9.3	1755.2 ± 61
63	F390A	−92.1 ± 1.2	−55.4 ± 2.9	−190.7 ± 19.2	1.4 ± 4.2	1727.9 ± 26.5
64	R393A	−106.3 ± 1	−54.5 ± 3.4	−201.1 ± 10.8	−11.5 ± 3	1715.3 ± 43.5
65	N61A	−101.3 ± 3.7	−54.7 ± 1.9	−191.1 ± 10.3	−8.4 ± 3.1	1730.6 ± 45.6
66	K68A	−98.9 ± 6	−56.2 ± 3.8	−182.1 ± 7.7	−6.2 ± 5.3	1741.2 ± 56.9
67	L97A	−94.9 ± 9	−56.6 ± 3.8	−176.9 ± 10.6	−2.9 ± 6.7	1718 ± 50.5
68	Q101A	−95.8 ± 7	−54.5 ± 3.6	−186.8 ± 31.2	−3.9 ± 8.9	1736.7 ± 22.9
69	W349A	−97.8 ± 3.5	−55.2 ± 2.5	−194.5 ± 11.1	−3.7 ± 5	1732.4 ± 17.7

Table includes energetic calculations with HADDOCK. vdW–van der Waals interactions; Elec–electrostatic interactions; Desol–desolvation energy; BSA–buried surface area; a.u.–arbitrary units of energy. *mutations performed on wild-type alanine residues (positive controls).

### Interface refinement

Heterodimers of ACE2 variants and SARS-CoV-2 RBD were extracted from all three datasets and submitted to water refinement with the HADDOCK webserver v2.2^22^ as previously described^23,24^, with the goal to optimize interface geometry and energetics. Briefly, ACE2/RBD heterodimers without glycans but in the presence of Zn^2+^ were uploaded to the HADDOCK refinement interface and submitted with default parameters. Weighting for the sorting of structures (scoring) after water explicit refinement^25–27^ were set for van der Waals energy (E_vdW_), Electrostatic (Coulombic) energy (E_elec_), Buried Surface Area (BSA), Interaction energy (dE_int_) and Desolvation energy (E_desolv_) to 1.0, 0.2, 0.0, 0.0 and 1.0, respectively^25^.

## Data Records

### Figshare and SBGrid

Structure files and associated data of human ACE2 variants in complex with SARS-CoV-2 RBD generated in this work have been deposited in Figshare^28^. The same data have also been deposited in SBGrid^29^.

Two folders are shared, (a) *6M0J* for the models derived from the crystal structure^30^ and (b) *6M17* for the models derived from the cryo-EM structure^11^. In each folder the following subdirectories are placed: *variants*, *ALA_scan*, and *UniProt*, and specifically for *6M17*, an additional subdirectory is included, *PyMOL_models_6M17*. This directory includes* .pdb* and* .cif* files of variants which were created by considering the complete cryo-EM model, with cofactors (ions, sugars) and all interfaces. In addition, the initial *.pdb* files that were used to produce all reported variants are placed in each folder (*6M0J_chains_AE.pdb* or *6M17_chains_BE.pdb*).

For the common subdirectories (*variants, ALA_scan*, *UniProt*), structure is as follows: The subdirectory *variants* contains data for ACE2 residue variants naturally occurring in the human population^16^, *UniProt* contains data for variants with *in vitro* mutations reported in the literature^17–20^, and *ALA_scan* contains data for variants resulted from the performance of computational alanine scanning mutagenesis at the interface of SARS-CoV-2 RBD and the human ACE2 receptor.

In detail, each subdirectory (*variants*, *ALA_scan*, *UniProt*) includes three files: the results file after the HADDOCK refinement^22^ (*.html* file), the parameter file that was used for the structure calculation (.*web*), and the top scoring refined structure file (*.pdb* file).The user can reproduce any run by uploading the*.web* file using the online server (https://haddock.science.uu.nl/services/HADDOCK2.2/haddockserver-file.html).

The nomenclature of each file in subdirectories *variants, ALA_scan* and *UniProt* corresponds to *XXXX_R1NUMR2*. *XXXX* stands for the *PDB ID* from which the model was extracted, *R1* is the one-letter residue code of the native residue of the ACE2 receptor, *NUM* is the residue number according to the Uniprot sequence of human ACE2 receptor and *R2* is the one-letter residue code of the variant to which the residue *R1* was changed. Results of the energetic calculations with HADDOCK for each generated variant of the complex are summarized in Online-only Table 1, and Tables 1 and 2.

### Github

An online structure viewer of the resulting models from all refinement runs and their energetics is available at: https://kastritislab.github.io/human-ace2-variants/. The structure viewer allows the user to visualize interface contacts, compare structural information, and be informed about the corresponding energetics for any model reported in this work.

## Technical Validation

### Data redundancy and structural mapping

Variants in the 3 datasets are distinct, showing minor overlap in terms of amino acid substitution (Fig. 2a). The computational alanine scanning shows a minor overlap with reported mutagenesis studies, where only 13% of the total mutations can be found in both datasets. In addition, only 1 out of the 39 *in vitro* designed ACE2 variants can be found in the human population (Fig. 2a). Mutations from missense variants are distributed across the entire ACE2 surface (Fig. 2b), including the interfaces with the SARS-CoV-2 RBD and B^0^AT1 partners^11^ (Fig. 2c). This structural mapping highlights the usefulness of ACE2 variants for structure-based design, as different residues affect the physical-chemical parameters of the receptor, and consequently, its underlying affinity towards different protein-protein interactions.

### Stereochemical quality

The stereochemical quality of derived models of ACE2 variants is of equivalent quality as their template structures, since we performed mostly single amino acid substitutions and refined them using restrained molecular dynamics simulations in explicit water^26^. This protocol is well-known to improve the quality of experimental structures and docking models^26,27^.

### Modeling from different templates

**Fig. 3:**
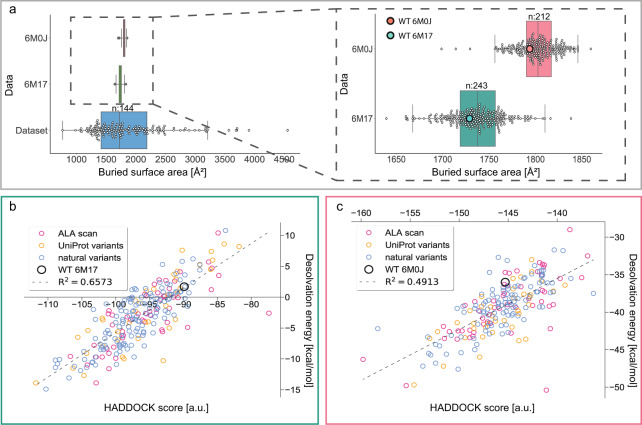
HADDOCK refinement of ACE2 variants in complex with the S-protein using the crystal structure (PDB ID: 6M0J) or the cryo-EM structure (PDB ID: 6M17) as reference. (**a**) On the left, calculation of buried surface area (Å^2^) for the crystal (top, 6M0J) and the cryo-EM structure (middle, 6M17) for all mappable variants and their comparison to the BSA of transient protein-protein interactions with known binding affinities (bottom, Dataset)^24^. On the right, a zoom into the distribution of BSA of both derived benchmarks, highlighting the method-specific packing of the interface area. (**b**) Desolvation energy against HADDOCK score for all variants calculated using the cryo-EM structure as an initial model, showing a high contribution of this energy to the overall HADDOCK scoring. (**c**) The same as (B) but using the crystal structure of the complex as an initial structure for subsequent HADDOCK refinement.

To assess the consistency of the HADDOCK water refinement protocol, we additionally constructed homology models using the crystal structure of the ACE2 in complex with the SARS-CoV-2 RBD^30^. Although the root-mean-square deviation (RMSD) between the C_α_ atoms of the residues from the two calculated structures is low (RMSD = 1.054 Å), we observed high variability in rotamer states, in particular for interface residues. The buried surface area (BSA, Å^2^) of both structures is within the distribution of BSAs for transient protein-protein interactions with known affinities^24^ (Fig. 3a). Interestingly, the crystallographic structure and designed variants have larger BSA as compared to the cryo-EM counterparts (Fig. 3a). This is expected since structures determined by X-ray diffraction are more tightly packed due to the crystal state of the protein. In contrast, the cryo-EM interface is smaller, likely because the specimen was captured in vitreous ice and was free in solution. In addition, model building during cryo-EM map interpretation is performed within an averaged Coulomb electrostatic potential map, which may lead to low resolution or absent densities in flexible regions and, therefore, less tight interface packing.

### Consistency in energy calculations

Usage of these two templates for generating variants and performing energy calculations constitutes an independent test for the robustness of the refinement protocol. Overall, for all generated models, high values for the corresponding Pearson-product momentum correlation coefficients are observed for HADDOCK score and underlying desolvation energies (Fig. 3b–c). This shows that energetic components for the HADDOCK score in both structures have similar contributions, desolvation energy being the most dominant. Only favourable energies are calculated for the variants when using the crystallographic model as an initial structure (Fig. 3c), whereas both favourable and unfavourable energies are calculated for the variants using the cryo-EM model (Fig. 3b). This is due to the presence of both transmembrane and soluble domains of the ACE2 in the cryo-EM model, whereas the crystallographic model includes only soluble domains. Desolvation energies, therefore, reflect contributions of solvation in the structures, in the presence or absence of the transmembrane regions.

### Overlap with external datasets

**Fig. 4:**
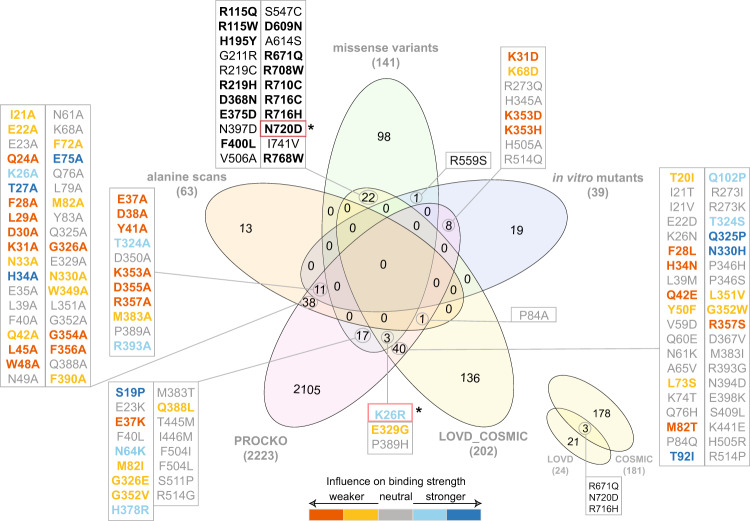
Venn diagram of datasets analyzed in this study. Overlap of datasets used for structural modeling (alanine scans, missense variants and *in vitro* mutants) with experimental deep scanning mutagenesis data (PROCKO^31^) and external datasets is shown. External datasets include open-access data deposited in LOVD 3.0 (https://www.lovd.nl) from COVID-19 patients and COSMIC^32^ from cancer patients. Overlapping mutations with *in vitro* determined interaction strength from deep scanning mutagenesis data^31^ are color-coded according to binding strength. Mutations in red boxes (N720D, K26R) are found in COVID-19 patients^35^. Mutations shown in bold font on the top represent common variants in both COSMIC and gnomAD ACE2 variants.
- ACE2 mutations from cancer patients derived from COSMIC v91^32^. Due to the higher risk of severe COVID-19 symptoms manifesting in cancer patients^33^, we have specifically focused on retrieving genetic variants of ACE2 available in COSMIC v91^32^ (Fig. 4). Interestingly, 15 genetic variants reported in gnomAD (R115Q, R115W, H195Y, R219H, D368N, E375D, F400L, D609N, R671Q, R708W, R710C, R716H, R716C, N720D, R768W) are also identified in cancer patients (Fig. 4, shown in bold). This result provides a hypothesis on the role of these mutations in SARS-CoV-2 infection to be further investigated.
- ACE2 mutations from COVID-19 patients included in LOVD 3.0^34^ (Fig. 4). LOVD 3.0 reports additional variants for the ACE2 receptor and includes the N720D mutation which has been identified as a variant in COVID-19 patients in the Italian population^35^. N720D is found in genomic data (gnomAD), cancer (COSMIC v91) and COVID-19 patients (LOVD 3.0). Another ACE2 protein variation identified in COVID-19 patients is the K26R, which is also included in the gnomAD data, but not in cancer patients. This mutation has been successfully expressed by Procko^31^ and appears to increase binding affinity for the RBD of the S protein **(**Fig. 4**)**. Interestingly, our respective 3D interaction model shows one of the lowest HADDOCK scores (−108.9 ± 5.1 a.u.), strongest van der Waals interactions (−57.8 ± 5.6 kcal.mol^−1^) and most favourable desolvation energy (−11.4 ± 7.9 kcal.mol^−1^) compared to all other analyzed mutations (Online-only Table 1). Considering the communicated correlation of HADDOCK score components with binding affinities for 144 protein-protein interactions^24^, the above-mentioned calculated energetic values corroborate the Procko results on the increased affinity for K26R, and therefore, possible higher infectivity of SARS-CoV-2. This is also corroborated by our distance calculations showing that K26R is only ~10 Å away from the interaction interface (Fig. 2).

To identify systematically present variations in our datasets, we overlapped the reported variations for which we communicate the respective structural models with 3 additional datasets described below:

- The experimental Procko dataset (PROCKO). A recent preprint tested affinity of 2,223 ACE2 missense mutants with the RBD of the S protein of SARS-CoV-2 after one round of selection^31^. Interestingly, overlap of those data with the 3 datasets described above is minor (78 common out of 242 mutations) (Fig. 4). In particular, overlap with genome variants is even lower (20 out of 141). This highlights the complexity underlying genome variation in the human population and the distinct evolutionary pressure of the ACE2 gene as compared to *in vitro* deep mutagenesis experiments. Still, our structural models for the 20 overlapping mutations which have available affinity values (S19P, E23K, K26R, E37K, F40L, N64K, M82I, G326E, E329G, G352V, H378R, M383T, Q388L, P389H, T445M, I446M, F504I, F504L, S511P, R514G) can act as a starting point for further characterization.

## Online-only Table

**Online-only Table 1:** Variants identified in human population, mappable on the cryo-EM structure.

#N	Variation	Haddock Score	vdW (a.u.)	Elec (a.u.)	Desol (a.u.)	BSA (Å^2^)
0	WT	−89.9 ± 5.2	−54.7 ± 2.4	−184.5 ± 9.3	1.7 ± 4	1729 ± 34.6
**1**	**E23K**	**−98 ± 11.1**	**−54.8 ± 3.3**	**−188.6 ± 24.2**	**−5.5 ± 8.8**	**1740.2 ± 62.6**
**2**	**K26R**	**−108.9 ± 5.1**	**−57.8 ± 5.6**	**−198.7 ± 10.8**	**−11.4 ± 7.9**	**1762.8 ± 43.4**
**3**	**E37K**	**−91.1 ± 5.2**	**−55.1 ± 2.2**	**−184.4 ± 17.5**	**0.9 ± 6.4**	**1737.9 ± 26.6**
**4**	**F40L**	**−99.5 ± 9.5**	**−57.2 ± 3.2**	**−187.5 ± 20.7**	**−4.8 ± 7.2**	**1763.7 ± 66.2**
5	S47P	−99.4 ± 3.5	−53.8 ± 3.3	−187.5 ± 14.3	−8.1 ± 3.9	1729.1 ± 41.3
6	N58D	−106.9 ± 6.2	−56 ± 3.6	−189.7 ± 39.9	−13.1 ± 6.2	1724.2 ± 16.8
7	N58H	−92 ± 8.1	−52.1 ± 4	−209.1 ± 10.1	2 ± 4.4	1739.9 ± 39
8	N64K	−103.4 ± 2.1	−53.9 ± 3.1	−195.1 ± 7.6	−10.5 ± 2.5	1746.1 ± 32.4
**9**	**M82I**	**−90.5 ± 6**	**−53.6 ± 3.4**	**−188.2 ± 19.5**	**0.8 ± 6.3**	**1708.7 ± 19.4**
10	Q86R	−93.2 ± 0.7	−55 ± 1.9	−198.7 ± 9.8	1.5 ± 2.5	1772.1 ± 23.6
11	A99T	−110.3 ± 7.5	−58.6 ± 3.1	−183.7 ± 16.5	−14.9 ± 4.3	1799.9 ± 28.7
12	N103H	−96.2 ± 1.5	−55.8 ± 2.3	−201.9 ± 15.5	0 ± 6	1713.3 ± 43.9
13	V107A	−94.9 ± 2.6	−54.6 ± 2.6	−204.8 ± 9.1	0.6 ± 3.8	1736.6 ± 18
14	R115Q	−99.1 ± 4.9	−56.9 ± 1.5	−205.7 ± 9.8	−1.1 ± 4.1	1766.2 ± 19.5
15	R115W	−100.1 ± 8	−54.9 ± 1.2	−192.3 ± 22.1	−6.7 ± 6.8	1742.6 ± 36
16	P138L	−108.3 ± 10.9	−54.8 ± 1.5	−217.4 ± 13.7	−10.1 ± 12.1	1735.6 ± 5.4
17	C141Y	−98.6 ± 2.9	−55.5 ± 1.9	−176.1 ± 10.7	−7.8 ± 3.5	1732.5 ± 35.8
18	N154K	−89.8 ± 3.5	−53.8 ± 0.7	−179.1 ± 12.2	−0.2 ± 6	1707 ± 13.2
19	N159S	−100 ± 9.5	−54.3 ± 2.1	−208.4 ± 15.2	−4.1 ± 7	1751.3 ± 50
20	E171D	−97.8 ± 4.2	−54.9 ± 0.9	−210.7 ± 19.4	−0.8 ± 4.9	1733.5 ± 56.3
21	V184A	−101.6 ± 7.4	−54.8 ± 2.9	−200.5 ± 10	−6.7 ± 10	1741.4 ± 45.1
22	A191P	−98.9 ± 3.5	−56.2 ± 2.2	−193.9 ± 9.5	−3.9 ± 5.2	1734.8 ± 68.5
23	H195Y	−89.4 ± 2.5	−56.6 ± 1	−156.2 ± 21.9	−1.5 ± 4.7	1748.7 ± 49.6
24	D206G	−97.5 ± 2.8	−56.9 ± 3.5	−191.4 ± 10.9	−2.3 ± 4.4	1745 ± 60.9
25	G211R	−103 ± 2.5	−58.5 ± 3.8	−204.2 ± 18.1	−3.6 ± 7	1816.3 ± 18
26	D216E	−95.9 ± 5.4	−55.2 ± 1.2	−174.3 ± 22.7	−5.8 ± 5.8	1731.3 ± 20.4
27	Y217C	−89.8 ± 2.9	−52.8 ± 2.2	−202.8 ± 6.2	3.6 ± 3.3	1729.9 ± 16.8
28	R219C	−101.5 ± 6	−52.6 ± 1.8	−215.2 ± 14.8	−5.8 ± 2.2	1748.4 ± 21.3
29	R219H	−98 ± 3.5	−56.4 ± 2.4	−207.1 ± 23.4	−0.2 ± 3.6	1759.8 ± 84
30	G220S	−99 ± 3.4	−56.6 ± 1.6	−191 ± 11.6	−4.3 ± 3.5	1771.6 ± 26.9
31	P235R	−97.7 ± 4	−54 ± 1.2	−187.3 ± 11.3	−6.3 ± 5.1	1685.9 ± 23.8
32	A251T	−97.6 ± 1.8	−55.8 ± 1.5	−209.1 ± 5.5	0 ± 0.9	1757.5 ± 9.2
33	Y252C	−94.5 ± 6.9	−52.8 ± 3.2	−179.7 ± 19.2	−5.8 ± 8.1	1711.6 ± 44.9
34	S257N	−93 ± 2.2	−56.6 ± 3.6	−186 ± 18.5	0.8 ± 1.5	1734.3 ± 27.6
35	P263S	−92.7 ± 6.3	−56 ± 2.7	−172.7 ± 31	−2.2 ± 4.7	1753.6 ± 37
36	M270V	−97.1 ± 1.7	−56 ± 2.1	−188 ± 5.5	−3.6 ± 1.6	1762.9 ± 44.2
37	V283F	−88.6 ± 2.4	−54.6 ± 4.8	−182 ± 12.9	2.4 ± 6.9	1739.8 ± 32.8
38	K288T	−92.1 ± 2.6	−56.2 ± 2.4	−182.3 ± 14.5	0.5 ± 2.4	1719.6 ± 30.4
39	I291K	−93.8 ± 2.7	−53.5 ± 2.9	−192.4 ± 17	−1.8 ± 4.2	1732 ± 15.6
40	D292V	−92.1 ± 1.3	−56.2 ± 2.1	−178.5 ± 19.3	−0.2 ± 1.7	1715.7 ± 23.4
41	D295G	−96.7 ± 9	−57.2 ± 2.2	−166.4 ± 30.9	−6.2 ± 7.5	1759 ± 30.2
42	M297L	−98.9 ± 8	−56.6 ± 2.2	−173.1 ± 5.4	−7.7 ± 7.7	1699.3 ± 7
43	M297I	−83.8 ± 7	−55.4 ± 1.2	−195.9 ± 9.9	10.8 ± 7.9	1715.3 ± 9.1
44	V298A	−87.6 ± 4.6	−55.9 ± 3	−180.4 ± 34.9	4.4 ± 8.2	1714.8 ± 47.3
45	D299G	−98 ± 2.4	−53.7 ± 2.4	−206.3 ± 17.3	−3 ± 3.3	1739.8 ± 44.7
46	E312K	−95.7 ± 2.7	−57.4 ± 3.1	−186 ± 18.4	−1.1 ± 1.8	1794.4 ± 28.3
**47**	**G326E**	**−103.3 ± 4.8**	**−58.1 ± 1.1**	**−216.1 ± 10.3**	**−2 ± 6.2**	**1838.4 ± 42.8**
**48**	**E329G**	**−91.2 ± 4.5**	**−56.8 ± 4.7**	**−175.5 ± 11.2**	**0.7 ± 5.5**	**1721.3 ± 22.3**
49	M332L	−98.1 ± 1.1	−54.4 ± 2.7	−194.6 ± 4.2	−4.8 ± 1.5	1760.8 ± 35.6
50	T334M	−93.9 ± 5	−54.6 ± 2.4	−195.6 ± 19.2	−0.2 ± 4.7	1761 ± 65.1
51	N338S	−99.1 ± 5.7	−56.8 ± 3.6	−180.1 ± 3.8	−6.3 ± 8.3	1751.2 ± 36.4
52	K341R	−95.6 ± 5.9	−55 ± 0.8	−210.1 ± 13.5	1.4 ± 4.6	1760.2 ± 23.4
**53**	**G352V**	**−108.6 ± 4.6**	**−58.3 ± 2.6**	**−197.8 ± 16.1**	**−10.8 ± 4.7**	**1792.5 ± 38.4**
54	D368N	−102.1 ± 2.3	−56.1 ± 5	−189.2 ± 5.3	−8.1 ± 3.9	1721.9 ± 43.3
55	E375D	−92.8 ± 2.2	−51.8 ± 2.7	−201.4 ± 19.4	−0.7 ± 4.3	1721.6 ± 30.2
56	M376T	−97.7 ± 2.8	−54.2 ± 3.1	−178.5 ± 22.3	−7.8 ± 3.2	1701.4 ± 45.8
57	G377V	−97.5 ± 4.2	−55.2 ± 2.8	−200.2 ± 18.3	−2.3 ± 5.3	1755.8 ± 35.5
58	H378R	−96.8 ± 4.7	−54.1 ± 3	−187.8 ± 14.3	−5.1 ± 6.4	1715.6 ± 8.4
**59**	**M383T**	**−96.5 ± 5.4**	**−55.5 ± 2.1**	**−185.8 ± 7.6**	**−3.9 ± 6.6**	**1697.7 ± 25.1**
**60**	**Q388L**	**−106.1 ± 2.7**	**−56.6 ± 2.6**	**−191.2 ± 23.1**	**−11.2 ± 3.9**	**1753.2 ± 21.4**
**61**	**P389H**	**−90.4 ± 5**	**−54.5 ± 1.4**	**−184.2 ± 4.8**	**0.9 ± 5.1**	**1753.4 ± 57.2**
62	N397D	−98.7 ± 5.9	−55.6 ± 1	−213.1 ± 26.9	−0.5 ± 6.7	1712.5 ± 58.7
63	F400L	−94.7 ± 5.4	−55.3 ± 2.5	−194.2 ± 23.5	−0.6 ± 8.1	1747.9 ± 10.7
64	L410V	−102.3 ± 5.9	−56.5 ± 2.2	−191.2 ± 3.1	−7.6 ± 7	1766.1 ± 14.7
65	L418S	−97.5 ± 5.5	−55 ± 4	−194.5 ± 18.4	−3.6 ± 10.5	1740.7 ± 55.9
66	S420C	−97.6 ± 3.9	−54.9 ± 2.4	−212.7 ± 8.8	−0.2 ± 4.9	1760.2 ± 16.6
67	S420P	−97.6 ± 7.9	−54.7 ± 3.8	−220.5 ± 8.1	1.2 ± 5.7	1751.4 ± 25.6
68	D427Y	−101 ± 4.2	−58 ± 2.2	−185.8 ± 15.8	−5.9 ± 4.9	1739.2 ± 54
69	N437S	−87.6 ± 3.5	−56.5 ± 3.1	−182.3 ± 15.7	5.3 ± 7.5	1738.5 ± 30.5
70	T445M	−99 ± 7.2	−55.8 ± 3.4	−198.1 ± 17.5	−3.6 ± 8.7	1756.9 ± 37.4
71	I446M	−91.8 ± 8.2	−54.4 ± 1.9	−189.4 ± 18.6	0.4 ± 12.6	1744.2 ± 102.6
72	V447F	−101.4 ± 7.7	−59.2 ± 4	−205.5 ± 21.3	−1.1 ± 3	1772.5 ± 52.4
73	G448E	−97 ± 3.1	−53.9 ± 3.3	−209.2 ± 20.8	−1.3 ± 4.7	1774.8 ± 62.9
74	M462I	−95.9 ± 5.1	−55.4 ± 0.8	−174.4 ± 23.8	−5.6 ± 7	1733 ± 39.7
75	E467K	−92.4 ± 1.6	−56 ± 0.8	−193.2 ± 10.5	2.2 ± 2.6	1757.3 ± 31.1
76	I468V	−94 ± 1.9	−55 ± 2.9	−182.3 ± 18.9	−2.5 ± 3.6	1705.7 ± 31.9
77	M480V	−96 ± 1.9	−55.1 ± 1.3	−199.5 ± 30.8	−1 ± 3.9	1730.8 ± 29.9
78	R482Q	−98.3 ± 1.4	−51.6 ± 2.6	−191.6 ± 16.8	−8.4 ± 6.8	1739.1 ± 46.2
79	E483D	−104.6 ± 6.6	−57.4 ± 2.4	−185.2 ± 25	−10.1 ± 8.3	1777.7 ± 24
80	P492S	−104.7 ± 2.3	−58 ± 4	−189.3 ± 10.3	−8.8 ± 2.6	1781.4 ± 34.9
81	D494V	−99.9 ± 2.6	−55.4 ± 3.5	−207.2 ± 13.2	−3.1 ± 8.1	1750.6 ± 15.8
82	A501T	−97.3 ± 2.1	−53.3 ± 1.4	−171.8 ± 25.3	−9.7 ± 4.8	1709.1 ± 23.6
83	F504L	−99.8 ± 5.8	−52 ± 1	−202.6 ± 4.9	−7.2 ± 5.8	1760.3 ± 28.6
84	F504I	−91.9 ± 1.5	−54.4 ± 4.3	−189.8 ± 6.5	0.5 ± 4.4	1726.4 ± 10.9
85	V506A	−89.8 ± 3.4	−54.6 ± 1.2	−184.2 ± 4.4	1.7 ± 4.6	1723.8 ± 22.2
86	S511P	−92.3 ± 1.4	−54 ± 2.7	−189.7 ± 31.2	−0.4 ± 9.6	1731.3 ± 46
87	R514G	−103 ± 3.9	−54.1 ± 2.2	−196.2 ± 8	−9.7 ± 5.3	1709.6 ± 24
88	F523L	−97.8 ± 5.7	−55.3 ± 2.4	−210.5 ± 10.6	−0.4 ± 4.8	1749.5 ± 60
89	A532T	−103 ± 3.9	−55.9 ± 1.8	−201.9 ± 8.1	−6.7 ± 3.5	1765.8 ± 40.5
90	K541I	−104.3 ± 8.7	−55 ± 3.5	−189.7 ± 34.8	−11.4 ± 5.1	1722.1 ± 41
91	N546D	−91.1 ± 3.1	−56.7 ± 2.3	−190.8 ± 21.4	3.8 ± 5.7	1744.5 ± 18.8
92	S547C	−93.9 ± 6.1	−52.9 ± 3.9	−197.2 ± 13.4	−1.6 ± 4.6	1743.6 ± 40.8
93	K553T	−97.2 ± 4.9	−57.4 ± 2.3	−205.9 ± 19.4	1.4 ± 4.7	1808.1 ± 36.6
94	R559S	−96.7 ± 0.7	−56.5 ± 0.9	−195.8 ± 7.3	−1 ± 2	1764.8 ± 57.3
95	S563L	−101.2 ± 5.6	−56.6 ± 3.2	−196.5 ± 18.8	−5.4 ± 6.5	1735.3 ± 46.4
96	L570S	−94.4 ± 5.5	−54.6 ± 3.7	−177.8 ± 18.2	−4.3 ± 1.9	1698.6 ± 19.2
97	R582S	−108.9 ± 4.7	−57.8 ± 1.2	−204.4 ± 10.2	−10.2 ± 3.2	1789.1 ± 34.8
98	R582K	−90.5 ± 6.1	−58.4 ± 2.6	−174.3 ± 3.8	2.8 ± 4.2	1734.7 ± 4.4
99	N586Y	−94.8 ± 3.3	−53.5 ± 2.6	−191.7 ± 6.8	−3 ± 4.2	1745.2 ± 32.4
100	T593N	−95.5 ± 2.6	−55.9 ± 0.8	−199.9 ± 19.9	0.3 ± 4.4	1742.9 ± 42.5
101	L595V	−93.1 ± 1.6	−57.4 ± 2.9	−204.9 ± 9.3	5.3 ± 4.5	1725.9 ± 55.1
102	D597E	−96 ± 4.4	−53 ± 4.4	−184 ± 16.1	−6.2 ± 7.7	1742.6 ± 73.1
103	S607G	−92.8 ± 1.5	−52.4 ± 3.1	−185.1 ± 8.5	−3.4 ± 1.7	1708.5 ± 46.4
104	D609N	−95 ± 1.9	−56.4 ± 1.2	−183.9 ± 12.6	−1.8 ± 3.8	1753.1 ± 33.6
105	A614S	−93.5 ± 2.3	−57 ± 2.4	−194.9 ± 28.1	2.4 ± 5	1777 ± 55.5
106	A614T	−103.5 ± 1.1	−53.9 ± 1.7	−195.1 ± 20.1	−10.6 ± 3.5	1746.8 ± 15.8
107	N638S	−101.8 ± 3.9	−53.8 ± 3.2	−197.3 ± 17.4	−8.5 ± 5.4	1748.8 ± 27.9
108	Y654S	−99.2 ± 6.8	−56.4 ± 2.5	−203.5 ± 10.8	−2.2 ± 5	1707.9 ± 14.3
109	E667V	−91.4 ± 3.6	−53.7 ± 1.6	−174.2 ± 23.1	−2.9 ± 7.2	1761.9 ± 30.6
110	V670A	−94.2 ± 4.8	−57.9 ± 1.5	−176.7 ± 16.3	−0.9 ± 6.4	1741 ± 48.6
111	R671Q	−99.9 ± 5.6	−54.9 ± 2.5	−170.7 ± 16.2	−10.9 ± 5.9	1711 ± 41.7
112	R671P	−98.8 ± 3.5	−52.7 ± 4.6	−192.6 ± 21.7	−7.5 ± 4.1	1728.5 ± 63.4
113	V672L	−99.4 ± 3.4	−54.1 ± 2.2	−182.4 ± 17.6	−8.8 ± 3.3	1737.4 ± 60.6
114	V672A	−94.7 ± 1.7	−55.9 ± 2.3	−195.2 ± 10.2	0.2 ± 5.3	1767.8 ± 41
115	A673G	−99.5 ± 10.9	−56.1 ± 2.2	−178.5 ± 47	−7.7 ± 9	1757.9 ± 58.2
116	K689E	−99.4 ± 4.3	−53.3 ± 1.3	−191.1 ± 23	−7.9 ± 4	1731 ± 50.3
117	S692P	−97.4 ± 7.3	−52.8 ± 2.5	−211.8 ± 17	−2.2 ± 4.1	1743.1 ± 41
118	D693H	−96.9 ± 7.1	−55 ± 2.2	−194 ± 23.1	−3.1 ± 5.9	1737.1 ± 27.7
119	P696T	−97.5 ± 7.1	−53.2 ± 1.6	−204.5 ± 7	−3.4 ± 6.1	1724.8 ± 32.5
120	R697G	−95.6 ± 3.2	−54.7 ± 2.4	−191.7 ± 11.3	−2.6 ± 1.5	1729.6 ± 26.5
121	V700I	−92.3 ± 5.1	−54 ± 0.9	−178.6 ± 11.1	−2.5 ± 3	1704.5 ± 45.7
122	R705S	−99.1 ± 4.7	−54.3 ± 4.8	−193.1 ± 14.2	−6.2 ± 7.8	1708.9 ± 23.1
123	R708W	−101.4 ± 4.9	−53.9 ± 4	−196.2 ± 11.3	−8.2 ± 3	1810.3 ± 38.6
124	S709R	−97 ± 4.1	−57.4 ± 3.1	−203.7 ± 2.9	1.2 ± 3.8	1763.4 ± 22.8
125	R710C	−98.5 ± 1.8	−55.8 ± 2.4	−202.2 ± 15.2	−2.3 ± 5.7	1791.5 ± 46.3
126	R710H	−95.8 ± 5.6	−53.5 ± 2.2	−172.2 ± 8.9	−7.9 ± 4.2	1694.5 ± 24
127	R716H	−105.2 ± 4.5	−54.1 ± 3	−195.6 ± 24.9	−11.9 ± 1.3	1761.4 ± 64.8
128	R716C	−96.1 ± 4.4	−56 ± 1.2	−177.5 ± 12.1	−4.6 ± 2.3	1725.1 ± 19.9
129	N720D	−97 ± 1.8	−53.7 ± 2.2	−189.3 ± 5	−5.5 ± 2.7	1743.2 ± 31.8
130	N720S	−102.2 ± 6.3	−54.5 ± 3.4	−196 ± 22.5	−8.5 ± 12.9	1783.9 ± 45
131	L722P	−99.9 ± 4.8	−55.6 ± 4.1	−175.3 ± 27.9	−9.2 ± 5.6	1724.6 ± 18.3
132	P729L	−88.6 ± 3.2	−57.5 ± 2.7	−186 ± 18.9	6.2 ± 6.3	1749.2 ± 45.5
133	L731F	−94.2 ± 1.7	−55.3 ± 3.8	−179.1 ± 19.1	−3.2 ± 1.8	1708.6 ± 47.4
134	S740F	−93.5 ± 4.1	−54.7 ± 2.3	−191.6 ± 20.5	−0.5 ± 6.3	1750.8 ± 25.1
135	S740P	−96.7 ± 4.5	−52.6 ± 5.6	−201 ± 12.8	−3.9 ± 6.4	1759 ± 49
136	I741V	−99.4 ± 11.5	−55.7 ± 3.7	−210.7 ± 6.9	−1.6 ± 10	1724.8 ± 73.3
137	V745I	−89.2 ± 2.1	−53.9 ± 2.7	−180.1 ± 6.3	0.7 ± 3.5	1710.7 ± 64.2
138	V755A	−101.6 ± 2.1	−56.2 ± 2.1	−188.1 ± 14.8	−7.8 ± 4.3	1739.8 ± 34.9
139	D767H	−90.2 ± 9.3	−57.2 ± 3.1	−193.8 ± 10.7	5.8 ± 7.3	1734.3 ± 31.7
140	R768W	−103 ± 5.4	−57.2 ± 2.5	−174 ± 40	−11 ± 7.8	1766.6 ± 28.5

Table includes energetic calculations with HADDOCK. vdW–van der Waals interaction score; Elec–electrostatic interaction score; Desol–desolvation score; BSA–buried surface area; a.u.–arbitrary units of energy. Bold entries include variants within 10 Å of the ACE2-S protein interface.
